# The threat of increased transmission of non-*knowlesi* zoonotic malaria in humans: a systematic review

**DOI:** 10.1017/S003118202300077X

**Published:** 2023-11

**Authors:** Rini Chaturvedi, Shibani Biswas, Kanika Bisht, Amit Sharma

**Affiliations:** 1 Molecular Medicine Group, International Centre for Genetic Engineering & Biotechnology, New Delhi, India; 2 Host–Parasite Biology, ICMR-National Institute of Malaria Research, New Delhi, India; 3 Academy of Scientific and Innovative Research (AcSIR), Ghaziabad, India

**Keywords:** host switching, non-human primate malaria infections in humans, non-*knowlesi* zoonotic malaria, zoonotic malaria

## Abstract

Of the 5 human malarial parasites, *Plasmodium falciparum* and *Plasmodium vivax* are the most prevalent species globally, while *Plasmodium malariae, Plasmodium ovale curtisi* and *Plasmodium ovale wallikeri* are less prevalent and typically occur as mixed-infections. *Plasmodium knowlesi*, previously considered a non-human primate (NHP) infecting species, is now a cause of human malaria in Malaysia. The other NHP *Plasmodium* species, *Plasmodium cynomolgi*, *Plasmodium brasilianum*, *Plasmodium inui*, *Plasmodium simium*, *Plasmodium coatneyi* and *Plasmodium fieldi* cause malaria in primates, which are mainly reported in southeast Asia and South America. The non-*knowlesi* NHP *Plasmodium* species also emerged and were found to cross-transmit from their natural hosts (NHP) – to human hosts in natural settings. Here we have reviewed and collated data from the literature on the NHPs-to-human-transmitting *non-knowlesi Plasmodium* species. It was observed that the natural transmission of these NHP parasites to humans had been reported from 2010 onwards. This study shows that: (1) the majority of the non-*knowlesi* NHP *Plasmodium* mixed species infecting human cases were from Yala province of Thailand; (2) mono/mixed *P. cynomolgi* infections with other human-infecting *Plasmodium* species were prevalent in Malaysia and Thailand and (3) *P. brasilianum* and *P. simium* were found in Central and South America.

## Graphical abstract

**Figure:**
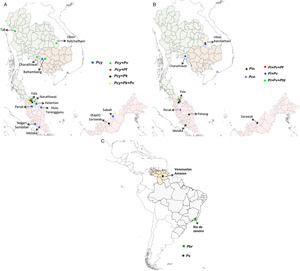

## Introduction

To date, only 5 confirmed *Plasmodium* species are known to be transmitted from one human host to another by *Anopheles* mosquitoes, namely, *Plasmodium vivax*, *Plasmodium falciparum*, *Plasmodium malariae*, *Plasmodium ovale wallikeri* and *Plasmodium ovale curtisi.* Globally, malaria elimination efforts are centred on 2 prevalent parasites: *P. falciparum* is the most lethal, while *P. vivax* is the most widespread species (Battle *et al*., 2019; World Health Organization, 2022). Other human malaria *Plasmodium* species, like *P. malariae* infection, are prevalent throughout the tropics and subtropics. *Plasmodium ovale curtisi* and *P. ovale wallikeri* are sympatric sibling species commonly found in sub-Saharan Africa and Asia (Sutherland *et al.*, 2010). The most common zoonotic malaria agent in Malaysia is *Plasmodium knowlesi*, the 6th species of human malarial parasite that was formerly believed to infect macaques but has since been found to infect humans (White, 2008; Muhammad *et al*., 2022; World Health Organization, 2022). *Plasmodium knowlesi* has also spread to some southeast Asian countries (White, 2008; Muhammad *et al*., 2022; World Health Organization, 2022). Although there have been no non-zoonotic malaria cases reported in Malaysia for the previous 4 years, there have been a total of 17 125 cases of *P. knowlesi* since 2017 (World Health Organization, 2022).

**Figure 1.**
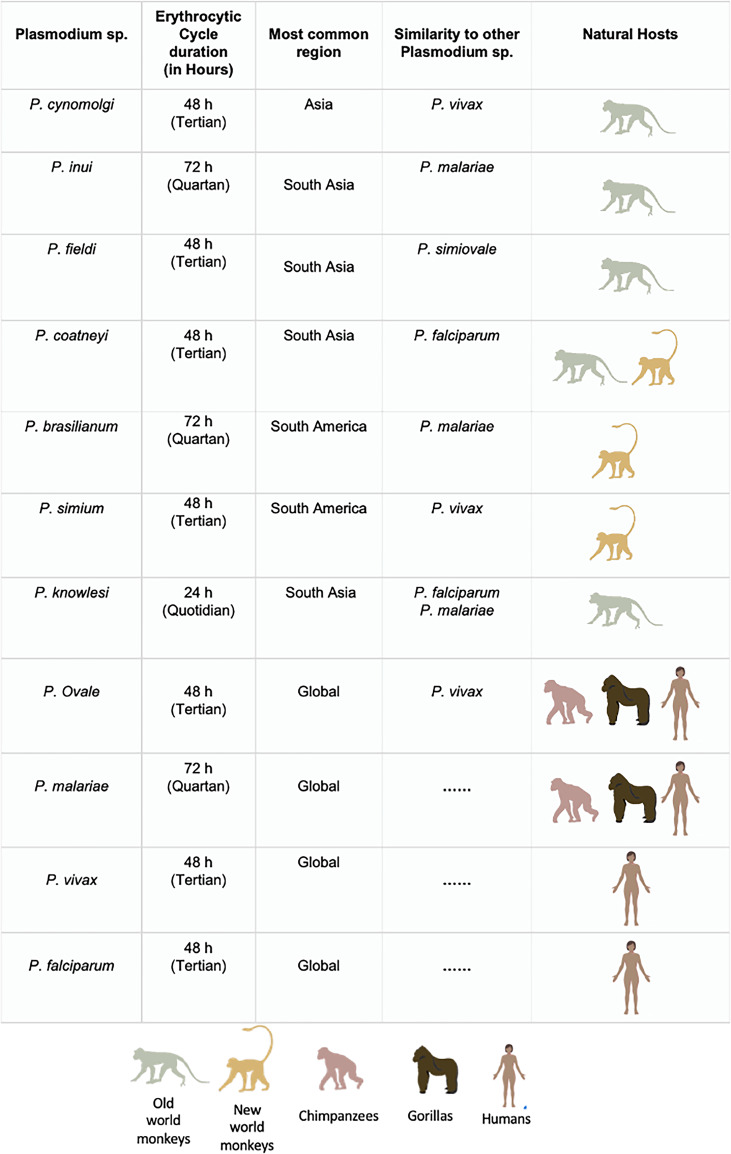
Non-human and human malaria primates considered in this study: erythrocytic cycle, their natural hosts, most common regions where the infections are reported from; similarities to other humans and their natural hosts. The details for natural hosts for *Plasmodium* species are adopted from Carlton (2018) and Escalante and Pacheco (2019).

The evolution and origin of *P. falciparum*, the most virulent parasitic species in the *Plasmodium* genus, has been the subject of intense research and discussion for many decades. Recent reports suggest that both *P. falciparum* and *P. vivax* evolved from wild-living African apes, as demonstrated by whole genome sequencing (Loy *et al*., 2017). *Plasmodium vivax* originated from an ancestral stock of parasites that infected gorillas, chimpanzees and humans in Africa, while *P. falciparum* emerged from a cross-species parasite transmission from gorillas (Loy *et al.*, 2017). A recent study illustrated that genome sequencing results of *P. malariae* that infects chimpanzees have similar selection characteristics to another *Plasmodium* lineage that can infect human and chimpanzee hosts (Fig. 1) (Rutledge *et al*., 2017). A human *P. ovale* variant has also been discovered in African apes, demonstrating a natural cross-species exchange of *P. ovale* infections between chimpanzees and humans (Fig. 1) (Duval *et al*., 2009; Rutledge *et al*., 2017).

Further, *P. knowlesi* infections are primarily zoonotic infections with wild macaques as their reservoir hosts, which could have adapted to switch their hosts to humans as the preferred hosts due to increasing human population and ecological changes (Fig. 1) (Lee *et al*., 2011). These studies suggest that malaria was a non-human primate (NHP) disease and that *P. vivax* and *P. falciparum* emerged as human infective agents in Africa with subsequent host-switching from gorillas (Loy *et al*., 2017). NHPs infecting *Plasmodium* species like *Plasmodium cynomolgi*, *Plasmodium simium*, *Plasmodium inui*, *Plasmodium brasilianum*, *Plasmodium coatneyi* and *Plasmodium fieldi* are among the zoonotic malaria species that have obtained the ability to infect humans *via Anopheles* (Sharp *et al*., 2020). In this study, we reveal trends in the emergence of NHP *Plasmodium* species and the global reports of their transmission to humans.

## Materials and methods

### Inclusion and exclusion criteria

We included only reports of NHP malaria infections in humans (either mono- or mixed-infections with any *Plasmodium* species) in humans with available full text. The exclusion criteria were: (1) studies including NHP *Plasmodium* species infection in their related natural hosts (Old and New World monkeys, chimpanzees and gorillas), (2) studies where sufficient reports could not be retrieved, (3) *P. knowlesi* infection reports in humans and (4) articles not available in English.

### Information sources and search strategy

**Figure 2.**
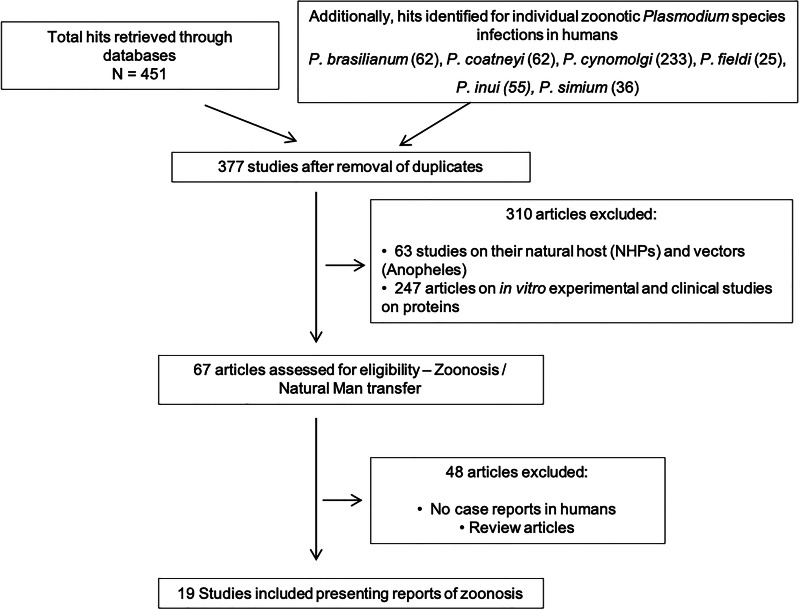
Flowchart depicting the study design for natural infections in humans by NHP *Plasmodium* species.

A systematic literature analysis was performed for all NHP malaria infections in humans. The data were collated and reviewed from the relevant literature that reports the cases of zoonotic *Plasmodium* infection transmission in humans from 2 search engines, PubMed and Medline. The terms used in the search were ‘rare *Plasmodium* species infecting humans’, ‘non-knowlesi zoonotic malaria’, ‘host switching’ and ‘macaque or non-human primate malaria’. Following this, we also used keywords for individual zoonotic ‘Plasmodium sp. (*P. cynomolgi*, *P. brasilianum*, *P. inui*, *P. simium*, *P. coatneyi*, and *P. fieldi*) human infections’, so no studies are missed for zoonotic human infections (Fig. 2). The time frame was not defined to check the initial reports of NHP infections in humans.

### Study selection and data extraction

Two independent reviewers reviewed titles and abstracts to collect publications that matched the inclusion criteria. The entire text of the publications was retrieved and evaluated for eligibility if the title and abstract of the paper could not be rejected with certainty by both researchers. Tables 1 and 2 display the lists of NHP infection cases in humans.

**Table 1.** Details of *Plasmodium cynomolgi* infections in humans

Species	Year	Samples (*n*)	Human malaria positive, *N* (%)	Mono-infection (*n*)	Mixed-infection (*n*)	Country	Location	Reference
*P. cynomolgi*	1960^a^	4		4		USA	Laboratory of Parasite Chemotherapy, NIAID	Eyles *et al.* (1960)
	1962^b^	5		5		USA	NIH, Bethesda, MD	Kuvin *et al.* (1962)
	1962–1963^b^	1		1		Cambodia and Malaysia	Institute for Medical Research, Kuala Lumpur, Malaysia	Bennett and Warren (1965)
	2007–2018	1359	1180	0	*P. cynomolgi* *+* *P*lasmodium *vivax –* 1	Thailand	Tak	Putaporntip *et al.* (2021)
	2007–2018			0	*P. cynomolgi* *+* *P. vivax* – 1	Thailand	Ubon ratchathani	
	2007–2018			0	*P. cynomolgi* *+* *P. vivax –* 1	Thailand	Chanthaburi	
	2007–2018			0	*P. cynomolgi* *+* *P. vivax −* 3; *P. cynomolgi* *+* *P. vivax* *+* *Plasmodium knowlesi* – 1; *P. cynomolgi* *+* *Plasmodium falciparum –* 1	Thailand	Yala	
	2007–2018			0	*P. cynomolgi* + *P. vivax* – 1	Thailand	Narathiwat	
	2011	–	1	1	0	Malaysia	Hulu Terengganu	Ta *et al.* (2014)
	2011–2014	61	55	5	0	Malaysia	Perak	Yap *et al*. (2021)
	2011–2015	163	13	2	0		Negeri Sembilan	
	2011–2016	32	13	1	0		Melaka	
	2011–2014	32	9	1	0		Kelantan	
	2013–2016	14 732	1361	11	*P. cynomolgi* + *P. vivax* – 2	Cambodia	Battambang	Imwong *et al*. (2019)
	2013–2017	–	1047	0	*P. cynomolgi* + *P. knowlesi* – 6	Malaysia	Kapit, (Sarawak)	Raja *et al*. (2020)
	2015	876	54	2	0	Malaysia	Northern Sabah	Grignard *et al*. (2019)
	2018	–	1	1	0	Thailand	Multiple locations travelled	Hartmeyer *et al*. (2019)
	2008 and 2009	5271	358	0	*P. cynomolgi* *+* *P. vivax –* 2	Thailand	Chanthaburi	Putaporntip *et al.* (2022)
	2008–2010		152	0	*P. cynomolgi* *+* *P. vivax –* 2		Narathiwat	
	1996; 2007–2009; 2013		923	0	*P. cynomolgi* *+* *P. vivax –* 1		Tak	
	2009–; 2013–2016		639	0	*P. cynomolgi* *+* *P. vivax* – 3		Ubon ratchathani	
	2007–2011; 2016–		2123	2	*P. cynomolgi* *+* *P. vivax* – 8; *P. cynomolgi* *+* *P. falciparum* – 2; *P. cynomolgi* *+* *P. knowlesi* – 2		Yala	
	2021	3	3	2	*P. cynomolgi* + *P. vivax* – 1	Thailand	Yala	Sai-ngam *et al.* (2022)

a Accidental infection of B strain of *P. cynomolg*i in human.

b Experimental transmission of *P. cynomolgi* in humans.

**Table 2.** Details of *Plasmodium inui*, *Plasmodium coatneyi*, *Plasmodium brasilianum*, *Plasmodium simium* and *Plasmodium fieldi* infections in humans

Species	Year	Samples (*n*)	Human malaria positives, *N* (%)	Mono-infection (*n*)	Mixed-infection (*n*)	Country	Location	Reference
*P. brasilianum*	2015	–	633	12	0	Venezuela	Venezuelan Amazon	Lalremruata *et al*. (2015)
*Plasmodium malariae*/*P. brasilianum*	2012–2013	4	3			Costa Rica	San José (Talamanca), Limón (San Carlos), Alajuela	Calvo *et al.* (2015)
*P. coatneyi*	2011–2014	61	55	3	0	Malaysia	Perak	Yap *et al*. (2021)
*P. fieldi*	2021	5271	2123	0	*P. inui* *+* *P. fieldi* *+* *P. vivax –* 3	Thailand	Yala	Putaporntip *et al.* (2022)
*P. inui*	2011–2014	32	13	1	0	Malaysia	Melaka	Yap *et al*. (2021)
		199	7	2			Sarawak	
	2020	71	–	2	0	Malaysia	Pahang	Liew *et al*. (2021)
	2008 and 2009	–	358	0	*P. inui* *+* *P. vivax* – 3	Thailand	Chanthaburi	Putaporntip *et al.* (2022)
	2008–2010	–	152	0	*P. inui* *+* *P. vivax* – 1; *P. inui* *+* *P. vivax* *+* *P. falciparum –* 1		Narathiwat	
	2009–; 2013–2016	–	639	0	*P. inui* *+* *P. vivax* – 1; *P. inui* *+* *P. vivax* *+* *P. falciparum –* 1		Ubon ratchathani	
	2007–2011; 2016–	–	2123	1	*P. inui* *+* *P. vivax* – 7; *P. inui* *+* *P. vivax* *+* *P. falciparum –* 1; *P. inui* *+* *P. fieldi* *+* *P. vivax –* 3		Yala	
*P. simium*	1966	1	1	1	*–*	Brazil	Sao Paulo	Deane *et al.* (1966)
*Plasmodium vivax*/*P. simium*	2001–2004	22	22	17	–	Brazil	Espírito Santo	Buery *et al.* (2017)
*P. simium*	2015–2016	–	49	28	0	Brazil	Rio de Janeiro	Brasil *et al.* (2017)
*P. simium*		9	9	8	–	Brazil	Atlantic Forest	De Alvarenga *et al.* (2018)

## Results

### Study identification and selection

**Figure 3.**
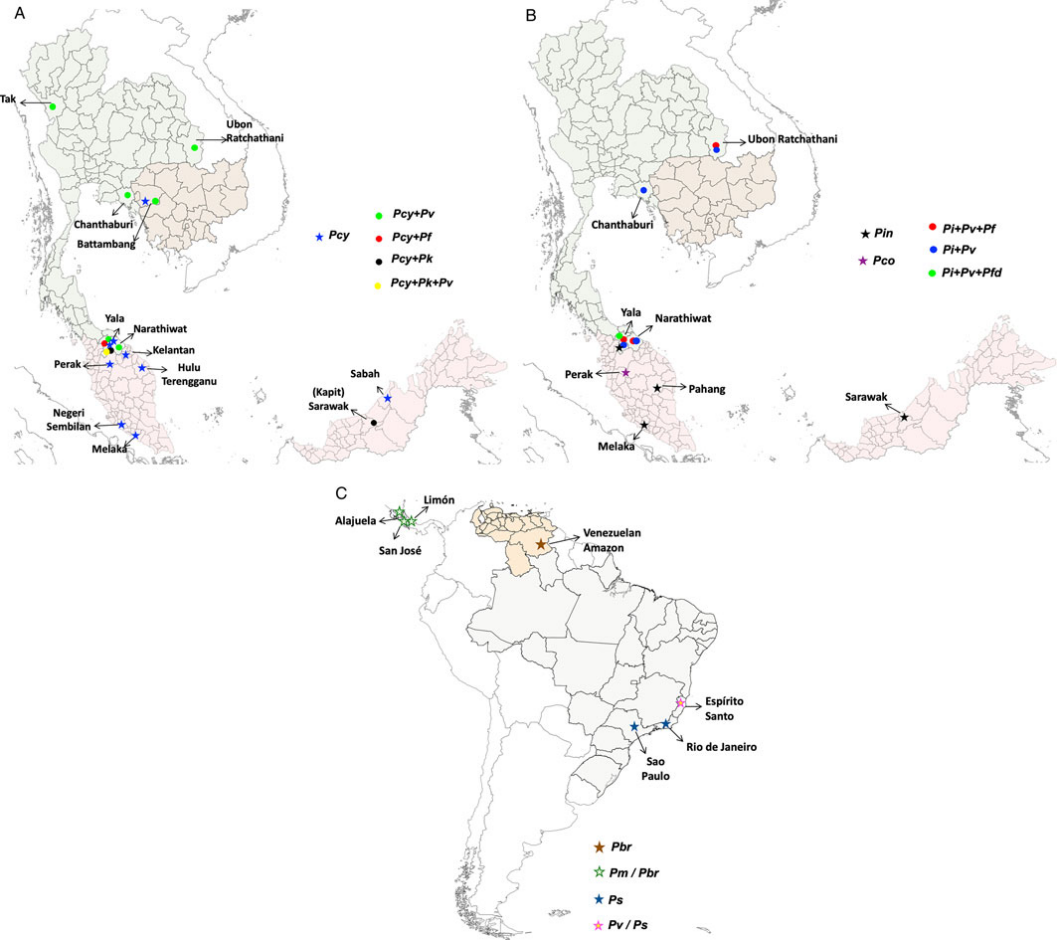
Locations of zoonotic *Plasmodium* species malaria infections in humans: (A) mono- and mixed-infections of *Plasmodium cynomolgi*, with *Plasmodium falciparum*, *Plasmodium knowlesi* and *Plasmodium vivax* in southeast Asian countries; (B) mono-infections of *Plasmodium inui* and *Plasmodium coatneyi*; mixed-infections of *P. inui* with *P. falciparum*, *Plasmodium fieldi* and *P. vivax* in southeast Asia and (C) mono-infections of *Plasmodium brasilianum* and *Plasmodium simium* in South America. The boundaries of countries are coloured as: Brazil (grey), Cambodia (light brown), Malaysia (pink), Thailand (light green) and Venezuela (nude). The shapefiles of the world map and the countries were downloaded from the University of Texas Libraries Geodata Portal (Hijmans, 2019).

We considered only non-*knowlesi Plasmodium* species, as *P. knowlesi* infections are already known to infect humans and have emerged as the dominant species in Malaysia (Muhammad *et al*., 2022). Nineteen studies from 377 search hits retrieved for NHP malaria infections in humans have been considered. Six NHP *Plasmodium* species with human transmission cases were individually included: *P. brasilianum*, *P. coatneyi*, *P. cynomolgi*, *P. fieldi*, *P. inui* and *P. simium*. Further, these 19 retrieved studies were also cross-checked by searching for individual studies for each *Plasmodium* zoonotic species, namely, *P. brasilianum*, *P. coatneyi*, *P. cynomolgi*, *P. inui*, *P. fieldi* and *P. simium* from 62, 62, 25, 233, 55 and 36 search hits, respectively. The spread of zoonotic species infections in humans is depicted in Fig. 3.

### Geographical distribution of zoonotic species to humans

#### *Plasmodium cynomolgi* zoonosis

*Plasmodium cynomolgi*, the most recent NHP parasite infecting humans, was cultivated *in vitro* in the early 1980s (Nguyen-Dinh *et al*., 1981). *Plasmodium cynomolgi* infections are predominantly found in macaque monkeys like *Macaca fascicularis* (long-tailed macaque) and *Macaca nemestrina* (pig-tailed macaque) (Fig. 1) (Chua *et al*., 2019). However, *P. cynomolgi* infections are also reported in experimental and rare natural zoonotic infections in humans (Eyles *et al*., 1960; Kuvin *et al*., 1962; Bennett and Warren, 1965; Garnham, 1966). The first recorded *P. cynomolgi* infection was in *M. fascicularis* in Indonesia in 1907, which was later acquired naturally by humans throughout southeast Asia from various macaque monkeys (Coatney *et al*., 1971; Kotepui *et al*., 2021). In terms of morphological and biological features, *P. cynomolgi* is nearly identical to its sister taxon *P. vivax* with its asexual replication, i.e. 48 h, the period between infection and the appearance of parasites in the blood (prepatent period), and the existence of a dormant stage (hypnozoites) (Fig. 1) (Cross *et al*., 1973; Most, 1973; Druilhe *et al*., 1980). Both *P. cynomolgi* and *P. vivax* prefer to infect reticulocytes and have Schuffner's dot-modified infected erythrocyte membrane (Bykersma, 2021). The experimental and accidental infection of humans with *P. cynomolgi* was shown half a century ago, with the suspicion that this simian parasite might infect humans and that an actual zoonotic outbreak would occur in the future (Cross *et al*., 1973; Most, 1973; Druilhe *et al*., 1980).

The first accidental *P. cynomolgi* (*P. cynomolgi bastianellii*) infection in humans was reported in the Laboratory of Parasite Chemotherapy, National Institute of Allergy and Infectious Diseases, while studying the *P. cynomolgi* subspecies in rhesus monkeys (*Macaca mulatta*). Four accidental infections by this simian malaria occurred among laboratory workers, proving that one particular ‘B strain’ of *P. cynomolgi* could produce malaria in humans (Eyles *et al*., 1960). The first naturally acquired *P. cynomolgi* human infection was reported in Hulu Terengganu, Peninsular Malaysia, in 2011, near a small forest crowded with macaques (Ta *et al*., 2014). The infection was initially diagnosed as *P. malariae*/*P. knowlesi* and later as *P. vivax* by microscopy and molecular methods. However, re-examination was performed *via* nested multiplex-polymerase chain reaction (PCR) followed by a parallel nested PCR for *Plasmodium* genus amplification, confirming the *P. cynomolgi* infection with asexual stages (Ta *et al*., 2014). In this region (Peninsular Malaysia), *Anopheles cracens* is the predominant mosquito species in Peninsular Malaysia and is hence suspected as a vector for the transmission of *P. cynomolgi*. A detailed survey conducted between 2011 and 2014 in 7 states of Malaysia identified 9 mono-infections of *P. cynomolgi* (8.8%) from multiple districts out of the 102 malaria-positive samples (Table 1) (Yap *et al*., 2021). This study also reported the infections of other zoonotic *Plasmodium* species: *P. coatneyi* and *P. inui*, with ~3% prevalence of 102 malaria-positive individuals (Table 1) (Yap *et al*., 2021). A malariometric study conducted in Cambodia between 2013 and 2016 reported that out of 1361 asymptomatic malaria-positive patients, 21 (~1.6%) were asymptomatic carriers of NHP malarial parasites (Imwong *et al*., 2019). Of these 21 patients, 52.4% of asymptomatic patients had *P. cynomolgi* mono-infections, while ~9.6% carried mixed-infections of *P. cynomolgi* and *P. vivax* (Imwong *et al*., 2019). From 2013 to 2017, a study of 1047 malaria-positive patients in Kapit, Malaysia, reported mixed-infections of *P. cynomolgi* and *P. knowlesi* in 6 clinical cases (~0.6%) in Sarawak, Malaysian Borneo (Raja *et al*., 2020). Another study conducted in 2015 in Sabah, Malaysia, identified 2 asymptomatic *P. cynomolgi* mono-infections (3.07%) out of 54 malaria-positive cases (Grignard *et al*., 2019). In Thailand during 2007–2018, out of 1180 symptomatic malaria patients reported *via* species-specific nested PCR, 9 were *P. cynomolgi* (0.76% prevalence) co-infections with *P. vivax* (0.59%), *P. falciparum* (0.09%) and *P. vivax* + *P. knowlesi* (0.09%) (Table 1) (Putaporntip *et al*., 2021). Most *P. cynomolgi* cases were reported in areas where macaques were in close proximity to humans (wild or domesticated). The study could not determine if *P. cynomolgi* caused symptomatic infections or coexisted asymptomatically with other human malarial parasites (Putaporntip *et al*., 2021).

Owing to the high prevalence of nocturnal mosquitoes and macaques, Thailand and similar regions may be a potential infection source for *P. cynomolgi* transmission to humans. In 2018, a traveller with a *P. cynomolgi* symptomatic infection was reported to have visited Thailand and Peninsular Malaysia (Hartmeyer *et al*., 2019). Another study conducted in Thailand in 2021 to probe simian *Plasmodium* species in blood samples of malaria patients reported 21 mono-infections with *P. cynomolgi* (Putaporntip *et al*., 2022). These 2 reports from Thailand highlight the occurrence of *P. cynomolgi* in diverse malaria-endemic areas of Thailand (Hartmeyer *et al*., 2019; Putaporntip *et al*., 2022). Further, Yala province in Thailand reported the highest number of mixed-infections (*n* = 17) of *P. cynomolgi* with other *Plasmodium* species (Fig. 3A) (Putaporntip *et al*., 2021, 2022). A recent systematic survey between 1946 and 2020 in southeast Asian countries compared the prevalence of *P. cynomolgi* infections in humans, mosquitoes and macaques in natural settings (Kotepui *et al*., 2021). The study demonstrated that the pooled proportion of naturally acquired *P. cynomolgi* was highest in macaques (47%), followed by mosquitoes (18%) and humans (1%) (Kotepui *et al*., 2021). Given this, *P. cynomolgi* transmission from mosquitoes to humans is likely constrained by the presence of macaque and *Anopheles* bite rates with their susceptibility (Kotepui *et al*., 2021).

#### *Plasmodium inui* zoonosis

*Plasmodium inui*, a quartan-type parasite, infects Old World monkeys with a typical 72 h quartan periodicity. *Plasmodium inui* natural hosts are macaques (*Macaca nigra*, *Macaca cyclopis*, *M. fascicularis*, *M. mulatta*, *M. nemestrina*, and *Macaca radiate*), and the primate infections are reported throughout Asia, including southern India, southeast Asia and Taiwan (Fig. 1; Table 2) (Eyles, 1963; Coatney *et al*., 1971). One vector responsible for *P. inui* transmission – *Anopheles leucosphyrus* (a vector of human malaria in Sarawak, Borneo) was identified in 1962, which was caught while biting a man, showing the possibility of transmission of a monkey infection to humans in nature (Coatney *et al*., 1971). Following this, 2 volunteers established the experimental natural transmission of *P. inui* to man via bites of infected mosquitoes (*Anopheles stephensi*/*Anopheles maculatus*) (Coatney *et al*., 1966). In 2020, an epidemiologic and entomological study from Pahang, Malaysia, revealed 2 natural, asymptomatic mono-infections of *P. inui* by nested PCR (Liew *et al*., 2021). All the tested individuals were participants who underwent forest training in 2020. The primers aimed at asexual and sexual 18S rRNA genes confirmed infection with *P. inui* (Liew *et al*., 2021). *Anopheles cracens* and *An. leucosphyrus* were shown to be the possible vector of monkey infection and transmission to humans in the wild. Both cases experienced minimal symptoms, and the parasitaemia was undetectable for short periods. Hence, the quartan *P. inui* parasite could be self-limiting in humans since it was not detected approximately 8 months after a patient's exposure to an infectious mosquito bite (Liew *et al*., 2021). According to a surveillance study, there was a 66.7% predominance of *P. inui* in all macaques tested in Pahang (26/39 macaques sampled), suggesting that humans may get infected with *P. inui via* vector-borne transmission from infected macaques to humans (Amir *et al*., 2020; Liew *et al*., 2021). In 2021, blood samples from malaria patients in 5 malaria-endemic regions of Thailand confirmed the natural transmission of *P. inui* in 19 patients out of all reported human and other infections (Putaporntip *et al*., 2022). Most patients infected with *P. inui* had concurrent infections with other *Plasmodium* species (Fig. 3B) (Putaporntip *et al*., 2022). Further, in 2011 and 2014, the blood samples from 14 villages in 7 states in Malaysia of indigenous populations of various sub-tribes recorded 3 *P. inui* infections while searching for *P. cynomolgi* cases (Table 2) (Yap *et al*., 2021). However, another study conducted from 2014 to 2015 from communities in Sarawak, Malaysian Borneo regions did not detect any *P. inui* and *P. cynomolgi* infections in humans (Siner *et al*., 2017). The locations of reported mono- and mixed-infections of *P. inui* in humans are shown in Fig. 3B.

#### *Plasmodium simium* zoonosis

*Plasmodium simium* was first identified in 1951 in the blood smear of *Alouatta clamitans* (brown howler monkeys) collected in the Atlantic Forest in Brazil (Fonseca, 1951; Deane *et al*., 1966). *Plasmodium simium* is found in south Brazil, where it is found among woolly spider monkeys (*Brachyteles arachnoids*), capuchin monkeys (*Cebus* and *Sapajus* sp.), arboreal howler monkeys (*Alouatta* sp.) and black-fronted titi monkeys (*Callicebus nigrifrons*) (Fig. 1) (Coatney *et al*., 1971). The first suspected natural infection of a human by *P. simium* was from Brazil (Deane *et al*., 1966). The infection was suspected on the basis that it had occurred in a forest reserve outside São Paulo where *P. simium* was known to be transmitted along with the morphological characteristics of the parasite (Deane *et al*., 1966). After half a decade, in 2015–2016, an epidemiological investigation of malaria patients in Rio de Janeiro, Brazil, reported a total of 28 mono-infections of *P. simium* out of 49 autochthonous malaria cases indicative of *P. simium* zoonosis in Brazil, although initially misdiagnosed as *P. vivax* (Fig. 3C; Table 2) (Brasil *et al*., 2017). The mitochondrial genome of these cases indicated that *P. simium* was most closely related to the South American *P. vivax* parasite (Brasil *et al*., 2017). The *P. simium* sequence revealed that it is similar to *P. vivax*, corroborating earlier claims that it originated from a host switch from humans to monkeys. Further, a study identified 7 distinct haplotypes from 22 human blood isolates (infected with *P. vivax* identified by microscopy) from Atlantic Forest inhabitants in Espírito Santo, Brazil (Fig. 3C). Of these 7 isolates, 2 isolates when shared with samples obtained from simians had an identical sequence to *P. simium* (Table 2) (Buery *et al*., 2017). This suggests that *P. simium* has been endemic in the Atlantic region but may have been incorrectly diagnosed as *P. vivax* due to the lack of any reliable diagnostic molecular techniques (Brasil *et al*., 2017; Buery *et al*., 2017). *Plasmodium simium* then adapted to the monkeys, and now occasionally infects humans in the region due to opportunistic infections (de Oliveira *et al*., 2021; Mourier *et al*., 2021). The primary vector in this area is suspected to be the *Anopheles (kerteszia) cruzi* which is found almost exclusively in the Atlantic region and can feed on both monkeys in the canopy and humans at the ground level (Deane *et al*., 1966, 196; Brasil *et al*., 2017). The isolates of *P. simium* from the New World monkeys and humans have a close genome-wide association with *P. vivax* from the New World. The genome-wide divergence between *P. simium* and New World *P. vivax* is negligible compared to intraspecific polymorphism in *P. vivax* populations of South America (de Oliveira *et al*., 2021). The differences between *P. vivax* and *P. simium* are focused on large deletions in the *P. simium* Duffy-binding protein 1 and reticulocyte-binding protein 2a genes which are usually present in all human-derived isolates (Mourier *et al*., 2021). There are only 2 unique single-nucleotide polymorphisms (SNPs) in the *P. simium* mitochondrial genome, differentiating it from *P. vivax* (Brasil *et al*., 2017; Rodrigues *et al*., 2018). Further, a recent study also identified 8 *P. simium-*specific SNPs out of 9 infected humans using an inexpensive tool specific to diagnose *P. simium* infections (De Alvarenga *et al*., 2018). Hence, it can be speculated that gene deletions in human-derived isolates and other genetic changes in the *P. simium* genome may have helped to invade human red blood cells (RBCs), thus explaining the basis of recent zoonotic infections (de Oliveira *et al*., 2021; Mourier *et al*., 2021).

#### *Plasmodium brasilianum* zoonosis

In New World monkeys, *P. brasilianum* is a simian parasite that causes quartan fever (Fig. 1) (Contacos *et al*., 1963). *Plasmodium brasilianum* was identified in at least 35 species of New World primates in Central and South America (Chaves *et al*., 2022). The genetic and morphological characteristics of *P. brasilianum* are indistinguishable from those of *P. malariae* (Contacos *et al*., 1963; Fandeur *et al*., 2000). Thus, *P. brasilianum* and *P. malariae* may specialize in different hosts but remain members of the same quartan malarial species. This anthropozoonotic parasite can easily circulate between humans or NHPs (Contacos *et al*., 1963). Investigations in the 1960s supported the theory that *P. brasilianum* could experimentally infect humans from monkeys and vice versa (Coatney *et al*., 1971). A comparison of the circumsporozoite protein and ribosomal small subunit (18S) in parasites of 75 *P. malariae*-positive patients revealed 16% with parasites that were nearly identical to the strain of *P. brasilianum* of infected monkeys from French Guiana (Fandeur *et al*., 2000; Lalremruata *et al*., 2015). These studies indicate that *P. brasilianum* is endemic to Latin America and that *P. brasilianum* and *P. malariae* parasites can spread quickly between humans and monkeys, serving as a natural reservoir for malaria (Lalremruata *et al*., 2015). The presence of *P. brasilianum* has already been established in howler monkeys in Central America (Costa Rica) (Chinchilla *et al*., 2006). Interestingly, a group identified 3 samples with 99% identity with *P. malariae*/*P. brasilianum* from human clinical samples in Costa Rica (Fig. 3C; Table 2) (Calvo *et al*., 2015). The analysis revealed a 99% identity with *P. malariae* isolated from atypical human cases in Asia, and a 99% identity with a sequence of *P. brasilianum* isolated from a non-human monkey of Guiana (Calvo *et al*., 2015). Similarly, another study demonstrated the genomic sequence identity of 99.70% in mitochondrial and apicoplast genomes of *P. brasilianum* with *P. malariae* (Talundzic *et al*., 2017). Additionally, it is established that while belonging to the radiation of human *P. malariae* strains, *P. brasilianum* does not represent a separate lineage and that *P. brasilianum* likely emerged after the human infection was transmitted to New World monkeys (Plenderleith *et al*., 2022).

#### *Plasmodium fieldi* zoonosis

*Plasmodium fieldi* asexual cycle is 48 h (Fig. 1). Bonnet macaques (*Macaca radiata*), long-tailed macaques (*M. fascicularis*), baboons (Papio doguera), rhesus macaques (*M. mulatta*) and pig-tailed macaques (*M. nemestrina*) are noted as natural hosts and reservoirs of *P. fieldi* (Eyles, 1963). In 2021, *P. fieldi* was reported to be capable of cross-transmission between macaques and humans under natural conditions (Putaporntip *et al*., 2022). While examining the symptomatic malaria patients in Thailand, a *P. fieldi* infection was diagnosed in 3 out of 5271 tested patients (Putaporntip *et al*., 2022). All *P. fieldi*-infected patients had concurrent infections with other *Plasmodium* species and responded well to chloroquine or artemisinin–mefloquine combination therapy (Table 2) (Putaporntip *et al*., 2022). A study conducted in Thailand determining the prevalence of different *Plasmodium* species in NHPs reported that of 93 macaque blood samples examined, *P. inui* (35%) and *P. fieldi* (30%) were the most prevalent species in malaria-positive macaques, presenting them as the natural reservoir and a potential public health concern to the local population (Fungfuang *et al*., 2020). The geographical distribution of reported *P. fieldi* infections in humans is depicted in Fig. 3B.

#### *Plasmodium coatneyi* zoonosis

*Plasmodium coatneyi* is commonly found in long-tailed macaques (*M. fascicularis*), and unlike other simian parasites, *P. coatneyi* shares morphological features to *P. falciparum* (Fig. 1) (Eyles, 1963; Fungfuang *et al*., 2020). A detailed study conducted between 2011 and 2014 in 7 states of Malaysia showed 3 mono-infections (2.17%) of *P. coatneyi* among 645 samples that tested positive for malaria (Table 2) (Yap *et al*., 2021). This study highlighted the existence of naturally acquired human infection with *P. coatneyi*, a species earlier believed to be incapable of infecting humans through infected monkey blood or mosquito bites (Yap *et al*., 2021). The geographical spread of *P. coatneyi* in humans is shown in Fig. 3B.

### Potential basis for transmission and their control

NHPs in their ‘forest habitat’, the Leucosphyrus-group *Anopheles* vectors, and human proximity to NHP habitats are linked to zoonotic malaria transmission. The ecology of mosquitoes' reservoir hosts and vectors is an important factor influencing the spread of zoonotic malaria to humans (van de Straat *et al*., 2022). In southeast Asia, the main species transmitting NHP malarial parasites and human-only malaria species in a few regions belong predominantly to the *An. leucosphyrus* (Moyes *et al*., 2014). From the perspective of public health, the Leucosphyrus subgroup is reported to be an effective vector, as numerous species have been implicated as zoonotic malaria vectors, including those for *P. knowlesi* (Collins *et al*., 1967, 1971). Traditional classifications of the *Anopheles dirus* and *An. leucosphyrus* complex species are considered forest and forest fringe inhabitants (Faust and Dobson, 2015). The interactions between humans, mosquitoes and monkeys, all of which are heterogeneous in space and time, are influenced by each group's behaviour. Infected mosquitoes must bite humans for zoonotic *Plasmodium* species to infect them. This necessitates being close to an infectious vector, which is often linked to shifts in land use, occupation and housing design (Ramasamy, 2014; Johnson *et al*., 2022). Additionally, humans and vectors that readily prey on humans and reservoir animals must be in proximity to the reservoir hosts or wildlife that harbours parasites. In their natural hosts, macaques, *P. knowlesi* and *P. cynomolgi* typically cause benign, long-lasting infections (Anderios *et al*., 2010). As a result, since the illness does not affect the monkeys' natural behaviours, infected monkeys make the best reservoirs for transmitting parasites to humans (Antinori *et al*., 2013).

The increasing reports of zoonotic/NHP *Plasmodium* species infecting humans within a decade are alarming. There might be multiple reasons for malaria transmission from primates to human by *Anopheles*. These might be (1) a growing need for more land for humans with the rapid increase in the human population in some regions that overlap with zoonotic infections; (2) substantial deforestation in tropical malaria-endemic countries (Kar *et al*., 2014); (3) increased contact between humans and mosquitoes that feed on NHPs along with increased interactions between humans and macaques due to urbanization and encroachment of NHP habitats (Kar *et al*., 2014); (4) the sequence analysis of NHP *Plasmodium* species underpins the genetic adaptations in the simian parasite that allow invasion of human RBCs and may explain the basis of recent zoonotic to human infections (Brasil *et al*., 2017) and (5) entomological factors such as vector distribution and potential, parasite prevalence and environmental patterns may also contribute to human transmission (Mills *et al*., 2010; Kar *et al*., 2014).

The use of indoor residual spraying and long-lasting insecticide-impregnated bed nets are 2 current mitigation strategies for reducing zoonotic malaria (World Health Organization, 2022). Nevertheless, these strategies might prove insufficient for other zoonotic control, as seen in *P. knowlesi* infections (Scott, 2020), since these strategies neglect the parasite's ongoing transmission between the host-animal reservoir populations.

There is no strong evidence for the efficacy of current antimalarial drugs against NHP parasite infections, but some studies reported the resolution of clinical symptoms with antimalarial drugs. *Plasmodium cynomolgi* infection was treated with atovaquone + proguanil followed by primaquine in the European traveller (Hartmeyer *et al*., 2019), artemether + lumefantrine followed by primaquine in Malaysian patients, and chloroquine + primaquine or artesunate + mefloquine in Thailand patients. Primaquine is prescribed as the primary antimalarial drug treatment because *of P. cynomolgi* parasite relapse; this must be made aware to the clinicians. Hence, the treatment of *P. cynomolgi* malaria shows a significant knowledge gap. A study in Thailand showed that *P. inui* and *P. fieldi* were responsive to chloroquine or artemisinin–mefloquine treatment (Putaporntip *et al*., 2022). In Brazil, *P. simium*-infected patients responded successfully to chloroquine and primaquine without hospital admission or relapses (Brasil *et al*., 2017).

## Discussion

Several studies conducted over half a century ago suggested that simian malaria could be transmitted to humans in experimental and accidental settings (Coatney *et al*., 1966; Deane *et al*., 1966; Cross *et al*., 1973; Most, 1973; Druilhe *et al*., 1980; Lalremruata *et al*., 2015). However, natural transmission of these (*P. cynomolgi*, *P. brasilianum*, *P. inui*, *P. simium*, *P. coatneyi* and *P. fieldi*) primate-related parasites to humans has been reported from 2010 onwards (Deane *et al*., 1966; Ta *et al*., 2014; Lalremruata *et al*., 2015; Brasil *et al*., 2017; Grignard *et al*., 2019; Hartmeyer *et al*., 2019; Imwong *et al*., 2019, 201; Raja *et al*., 2020; Liew *et al*., 2021; Putaporntip *et al*., 2021, 2022; Yap *et al*., 2021). Zoonotic malaria has been reported in humans in many parts of southeast Asia under natural conditions through the bite of infected *Anopheles* mosquitoes (Deane *et al*., 1966; Ta *et al*., 2014; Lalremruata *et al*., 2015; Brasil *et al*., 2017; Grignard *et al*., 2019; Hartmeyer *et al*., 2019; Imwong *et al*., 2019, 201; Raja *et al*., 2020; Liew *et al*., 2021; Putaporntip *et al*., 2021, 2022; Yap *et al*., 2021). Because of their proximity to monkey reservoir hosts and mosquito vectors, people who live on the forest periphery, work in or travel in forested areas are most likely to get zoonotic malaria (Kar *et al*., 2014; Lalremruata *et al*., 2015; Kotepui *et al*., 2021). The absence of highly sensitive techniques for detecting the parasites to differentiate between morphological and genetic similarities was a significant hurdle in correctly identifying *Plasmodium* species. To identify and confirm *Plasmodium* species, all reports included in the current study used either highly sensitive nested PCR or semi-nested for small subunit rRNA gene or cytochrome C oxidase gene amplification and sequencing. It is inferred that *P. cynomolgi*, *P. fieldi*, *P. simium* and *P. inui* infections are zoonotic transmissions that possibly originated and diverged from *P. vivax* while infections of *P. brasilianum* are from *P. malariae* (Sharp *et al*., 2020). Based on the cumulative studies, zoonotic malaria transmission has only occurred in South America and southeast Asian countries until now (Fig. 3). A close examination of epidemiology and parasite transmission also revealed that the distribution of zoonotic malaria cases depends entirely on *Plasmodium* species and on the demographics of the human host populace in different geographical locations (van de Straat *et al*., 2022). For example, most *P. cynomolgi*, *P. inui*, *P. fieldi* and *P. coatneyi* cases are reported in Asian countries, while *P. simium* and *P. brasilianum* cases are from South America (Fig. 3). Compared to other zoonotic cases, the proportion of mono-/mixed-infections caused by *P. cynomolgi* was more prevalent than other reported rare infections. The *P. cynomolgi* infections were reported from southeast Asian countries, majorly from Thailand, and it is alarming that the number of reported *P. cynomolgi* cases has increased in the past 10 years (2011–2020). In contrast, *P. simium* and *P. brasilianum* are endemic in South America (Brazil and Venezuela) (Fig. 3). Unlike other simian parasites, *P. fieldi* cases have been reported recently in 2021 from Thailand, mostly, and all *P. fieldi* co-exist with other malarial parasite species only (Imwong *et al*., 2019).

In contrast, *P. inui* infections in humans are widespread throughout southern Asia (Imwong *et al*., 2019; Yap *et al*., 2021). No mixed-infections have been reported with *P. simium*, *P. brasilianum* and *P. coatneyi* until now. So, unlike *P. cynomolgi* and *P. simium*, the rarely reported cases of *P. fieldi*, *P. inui* and *P. coatneyi* require additional future research efforts. The establishment and spread of these zoonotic species as human-infecting *Plasmodium* species highlight the importance of understanding how parasites' transmission capabilities adapt to new hosts, and to predict future zoonotic malaria outbreaks. It is clear that NHP malarial parasites are a potential reservoir of infectious human parasites. A molecular assessment of these infections is provided by malariometric studies of asymptomatic human infections with NHP parasites. These surveys typically use microscopy, rapid diagnostic tests or non-species-specific PCR, making it difficult to identify malarial parasites that infect NHPs. In this situation, whole-genome amplification and species-specific PCR will be beneficial. More surveillance studies and possible control measures should be considered to curtail the transmission of these parasites to achieve malaria elimination worldwide. Further, the increased encroachment of reservoir hosts by humans and the outdoor blood-biting habits of the vectors also pose challenges to mitigation efforts due to zoonotic malaria (Scott, 2020). Hence, a trans-disciplinary approach targeting vectors' contact with humans must be considered rather than the conventional malaria control methods. This can be accentuated by monitoring wild macaques and their carriers. It would be advisable to understand better the epidemiology of the *Plasmodium* parasites they harbour and develop effective strategies for minimizing the potential threat of zoonotic malaria infections.

## Data Availability

All data are included in the paper.
